# Tissue-resident memory CD103+CD8+ T cells in colorectal cancer: its implication as a prognostic and predictive liver metastasis biomarker

**DOI:** 10.1007/s00262-024-03709-2

**Published:** 2024-07-02

**Authors:** Shijin Liu, Penglin Wang, Peize Wang, Zhan Zhao, Xiaolin Zhang, Yunlong Pan, Jinghua Pan

**Affiliations:** 1 Department of General Surgery, The First Affiliated Hospital of Jinan University, Guangzhou, 510632 China; 2 Department of Pathophysiology, School of Medicine, Jinan University, Guangzhou, 510632 China; 3 Department of Gastrointestinal Surgery, The Fifth Affiliated Hospital of Jinan University, Heyuan, 517000 China; 4 MOE Key Laboratory of Tumor Molecular Biology and Key Laboratory of Functional Protein Research of Guangdong Higher Education Institutes, Institute of Life and Health Engineering, Jinan University, Guangzhou, 510632 China

**Keywords:** Tissue-resident memory CD103+CD8+ T cell, Colorectal cancer, Liver metastasis, Prognosis, Immunotherapy

## Abstract

**Background:**

Tissue-resident memory CD103+CD8+ T cells (CD103+CD8+ TRMs) are important components of anti-tumor immunity. However, the significance of CD103+CD8+ TRMs in colorectal cancer (CRC) and their advantages remain unclear.

**Methods:**

Clinical data and specimens were used to evaluate the significance of CD103+CD8+ TRMs in CRC. A mouse subcutaneous tumorigenesis model and colony-formation assay were conducted to evaluate the anti-tumor effects of CD103+CD8+ TRMs. Finally, the infiltration density and function of CD103+CD8+ TRMs in the tumors were evaluated using flow cytometry.

**Results:**

In this study, we showed that highly infiltrated CD103+CD8+ TRMs were associated with earlier clinical stage and negative VEGF expression in CRC patients and predicted a favorable prognosis for CRC/CRC liver metastases patients. Interestingly, we also found that CD103+CD8+ TRMs may have predictive potential for whether CRC develops liver metastasis in CRC. In addition, we found a positive correlation between the ratio of the number of α-SMA+ vessels to the sum of the number of α-SMA+ and CD31+ vessels in CRC, and the infiltration level of CD103+CD8+ TRMs. In addition, anti-angiogenic therapy promoted infiltration of CD103+CD8+ TRMs and enhanced their ability to secrete interferon (IFN)-γ, thus further improving the anti-tumor effect. Moreover, in vivo experiments showed that compared with peripheral blood CD8+ T cells, CD103+CD8+ TRMs infused back into the body could also further promote CD8+ T cells to infiltrate the tumor, and they had a stronger ability to secrete IFN-γ, which resulted in better anti-tumor effects.

**Conclusion:**

We demonstrated that CD103+CD8+ TRMs have the potential for clinical applications and provide new ideas for combined anti-tumor therapeutic strategies, such as anti-tumor angiogenesis therapy and CAR-T combined immunotherapy.

**Supplementary Information:**

The online version contains supplementary material available at 10.1007/s00262-024-03709-2.

## Introduction

Colorectal cancer (CRC) is one of the most common cancers and the third leading cause of cancer-related mortality in the world [1, 2]. The 5-year survival rate of patients with metastatic colorectal cancer (mCRC) is less than 20% [3, 4]. Many studies have shown that individualized treatments based on different molecular and clinicopathological characteristics of tumors can improve the prognosis of patients with CRC. However, the value of tumor molecular or clinicopathological characteristics such as programmed death-ligand 1 (PD-L1), microsatellite stability (MSI), and tumor mutational burden (TMB) is limited because of the heterogeneity of tumors and tumor microenvironment (TME), among other reasons [5, 6]. Therefore, there is still a need to explore the molecular or clinicopathological characteristics of tumors that are more accurately associated with the prognosis of CRC patients so that we can provide better ideas for the diagnosis and treatment of CRC.

Recently, tissue-resident memory CD8+ T cells (CD8+ TRMs) have been identified as a new subpopulation of CD8+ T cells. It has tissue-resident properties and an independent protective capacity that contributes to immune monitoring and immune responses in peripheral tissues [7, 8]. CD69 and CD103 are typical markers of CD8+ TRMs [9, 10]. In particular, CD103 is an important protein that allows CD8+ TRMs to reside in the epithelial tissue when it binds to E-cadherin. More importantly, this process also increases the binding strength between T cells and tumor cells, promoting the release of lysed particles and cytokines and enhancing the immune response and target cell killing [11, 12]. Overall, many studies have shown that the CD103+CD8+ TRMs subpopulation of T cells plays an important anti-tumor role [13, 14]. However, the extent of CD103+CD8+ TRMs infiltration in CRC tissues, its role and significance in CRC, and its potential for clinical application remain unknown.

In this study, we found that CD103+CD8+ TRMs are a new prognostic biomarker that can be used to evaluate the susceptibility of patients with CRC to liver metastasis. Preclinical experiments have demonstrated that CD103+CD8+ TRMs have better anti-tumor ability than normal CD8+ T cells, and anti-tumor angiogenesis treatment can promote their infiltration.

## Materials and methods

### Patient cohort and samples

Paraffin-embedded tissue specimens of CRC and matched adjacent normal tissues were obtained from 306 patients with CRC who attended the First Affiliated Hospital of Jinan University between January 2014 and December 2016. Matched adjacent normal tissue was defined as normal tissue 5 cm or more from the tumor margin and identified by a pathologist. Clinical data including age, gender, tumor location, T-stage, N-stage, M-stage, clinical stage, MMR status, expression of EGFR and VEGF, and the number of CD8+ T cells and CD103+CD8+ TRMs infiltration were collected (Supplementary Table 1). This study was performed in accordance with the Declaration of Helsinki and approved by the Medical Ethics Committee of the First Affiliated Hospital of Jinan University.

Follow-up information was obtained mainly from the hospitalized medical record information and telephone follow-up. Postoperative follow-up was until death or January 2022, with a median follow-up period of 52.5 (1.0–84.0) months. We observed the overall survival (OS) and progression-free survival (PFS) of patients. Follow-up information mainly included the basic condition of the patient, recurrence/metastasis, regression of the disease, and survival status. Tumors were staged and graded based on the 8th edition of the American Joint Committee on Cancer tumor-node-metastasis (TNM) staging system.

### Multiplex immunofluorescence (mIF)

Paraffin-embedded tissue specimens of CRC and matched adjacent normal tissues were sectioned at a thickness of 4 μm, and air-dried tissue slides were placed in a 65 °C baking oven for 1 h. The tissue slices were then de-waxed in xylene and hydrated in ethanol. Antigenic thermal repair was performed in Tris–EDTA buffer for 5 min and then left to cool to room temperature. The tissue slices were then covered with 0.5% TritonX-100 (prepared in PBS) for 20 min (permeabilization), 3% H_2_O_2_ for 25 min (blocking), and QuickBlock™ Immunostaining Confinement Solution for 30 min (closure). The slides were then incubated overnight with primary antibodies against CD103 (1:100; 95835S; CST), CD8(1:100; 70306S; CST), CD31(1:50; AF0077; Affinity Biosciences), and α-SMA(1:100; BM0002; BOSTER) at 4 °C. Then, TSA-488/546/647 staining working solution (Servicebio) was used to cover the tissues to be stained, incubated for 10 min at room temperature, and protected from light. According to the type of primary antibody, the slides were incubated at room temperature for 50 min with the corresponding secondary antibody (1:800; 7074/7076/7077; CST). Finally, the DAPI working solution was added dropwise and incubated for 10 min at room temperature, protected from light, and then sealed with an anti-fluorescence quenching agent. At least three fields (40× magnification) per area were evaluated for each marker. Then, the average number of CD8+ T cells and CD103+CD8+ TRMs was calculated, respectively, and then the best cut-off value was calculated using X-tile software.

### Evaluation of TRM and the normalization of tumor vasculature

The differentiation and development of TRM are strongly associated with CD103, and solid epithelial tumors (melanoma, breast cancer, etc.) are often infiltrated by cells expressing CD103+CD8+ TRMs [12, 15, 16]. Therefore, most studies, including this one, have also used CD103 to label TRMs. Thus, T cells co-expressing CD103 and CD8 were CD103+CD8+ TRMs, and their numbers were counted using ImageJ software. In addition, we used ImageJ software to count the number of α-SMA+, CD31+, and α-SMA+ CD31+ vessels. Then, the ratio of α-SMA-positive vessels to the sum of CD31 and/or α-SMA-positive vessels [α-SMA/(CD31+α-SMA)] was calculated, and its median was used as the cut-off value of the subgroups to divide the vascular status into normalized and non-normalized.

### Cells

MC38 cell lines were provided by the Cell Bank of the Chinese Academy of Sciences and were cultured in DMEM (Gibco) with 10% FBS (ExCell Bio) and 1% penicillin–streptomycin (Gibco). Mouse CD8+ T cells and CD103+CD8+ TRMs were cultured in complete RPMI 1640 medium supplemented with 10% FBS (ExCell Bio), 1% penicillin–streptomycin (Gibco), and IL-2 (100 IU/ml). All cell lines tested negative for Mycoplasma using the Mycoplasma Detection Set (M&C Gene Technology) at 37 °C in a humidified atmosphere containing 5% CO_2_.

### Mice

Forty-two C57BL/6 mice aged 5–7 weeks, weighing 18–26.5 g were provided by the Guangdong Medical Laboratory Animal Center. Mice were subcutaneously inoculated with MC38 cells. Mice were housed under standard conditions and observed daily. Tumors were allowed to grow. Tumor growth was observed in all 42 inoculated mice, indicating a 100% tumorigenesis rate. Tumor volumes were measured with a caliper using the length (L), width (W), and height (H) and calculated as tumor volume = L × W × H/2. Animals were considered dead when the tumor volume reached > 1500 mm^3^. Tumors that were too large or small were excluded. Forty mice were used in this study. Forty mice were divided into eight groups using the random number method, and the experiment was implemented according to the preset experimental plan. There were no differences in the average tumor volumes among the groups. On Day 21, all mice were sacrificed by neck dissection. All animal experiments were performed in accordance with the guidelines of the Ethics Committee for Animal Experiments at Jinan University. The Ethics Committee for Animal Experiments of Jinan University approved the study protocol.

### Isolation of mouse T cells

The mice were sacrificed by cervical dislocation. Mouse peripheral blood was extracted and passed through a cell sieve. In addition, the mice were aseptically dissected and the tumors were removed, sieved through a cell sieve, and crushed. After removing the supernatant, red blood cell lysis buffer was added and centrifuged to remove the supernatant. The cells were resuspended for culture and removal of insoluble tissue fibers, and then the cells in suspension were counted. The EasySep™ Mouse CD8+ T Cell Enrichment Kit (Stem Cell Technologies, 19,853) was used to isolate CD8+ T cells. Then, the suspensions of CD8+ T cells with a purity of more than 90% were obtained and subsequently analyzed by flow cytometry.

### Flow cytometry

Flow cytometry was used to isolate CD103+CD8+ TRMs and to assess the density of infiltrating CD103+CD8+ TRMs and the expression levels of interferon IFN-γ in the tumors. According to the above method, tumor-infiltrating CD8+ T cells and peripheral blood CD8+ T cell suspensions were obtained and viability assayed to be over 90%. And 1 μl of fluorescein isothiocyanate (FITC) anti-mouse CD8 (cat. 11-0081-81, eBioScience), phycoerythrin (PE) anti-mouse CD103 (cat. E-AB-F1090D, Elabscience), and APC anti-mouse IFN-γ (cat. E-AB-F1101UE, Elabscience) were added to the tube, respectively. Then, CD103+CD8+ TRMs were sorted out by flow cytometry and the infiltration density of CD103+CD8+ TRMs and the expression level of IFN-γ were assayed.

### T cell-mediated tumor cell killing assay

MC38 cells were cultured in advance in RPMI 1640 complete medium, and cells with normal proliferative capacity were retained. In 6-well plates, 1500 tumor cells were seeded per well and cultured for 10 days. MC38 cells were co-cultured with CD103+CD8+ TRMs or peripheral blood CD8+ T cells in a 3:1 ratio and stimulated with anti-mouse CD3 2.5ug/ml and CD28 2.5ug/ml. After 4 days of coculture of tumor cells and T cells in 12-well plates, wells were washed with PBS twice to remove the T cells, and the surviving tumor cells were fixed and stained with a crystal violet solution. The dried plates were scanned and quantified.

### In vivo drug treatment and adoptive transfer

Eight-week-old C57BL/6 J female WT mice were injected with 250,000 MC38 cells. To assess the effect of anti-angiogenic therapy on CD103+CD8+ TRMs, one group of mice was injected with bevacizumab at 15 a dose of on days 0, 2, 5, and 8. The same mixture without the drug was injected as a vehicle to control the animals. Depletion of CD8+ T cells was achieved by administering anti-mouse CD8β antibodies (cat. BE0223, BioXCell) at 250 mg dose at day 0, 2, 5, 8 and 12. As mentioned previously, CD103+CD8+ TRMs were isolated from congenically marked mice. To evaluate the anti-tumor capacity of CD103+CD8+ TRMs, 10,000 CD103+CD8+ TRMs, or peripheral blood CD8+ T cells were adoptively co-transferred into congenically marked C57BL/6 J mice prior to tumor implantation. Tumor volume was measured with a caliper every 2–3 days and calculated the tumor volume.

### Statistical analysis

Data were analyzed and processed using the statistical software GraphPad Prism 8.0. Normally distributed data are expressed as X ± s, and analyses with two groups (kindness intervention group and non-kindness intervention group) were subjected to t-tests. The X-tile software was used to obtain the best cut-off value [17]. The relationship of clinical data was analyzed using the Chi-square test (χ^2^ test), Kaplan–Meier survival analysis, log-rank test, and Cox risk regression analysis. Spearman’s correlation was used to analyze the α-SMA coverage rate and infiltration density of CD103+CD8+ TRMs in CRC tissues. Error bars display standard deviations (SD) unless otherwise noted. *p* values are noted as *p < 0.05; **p < 0.01; ***p < 0.001.

## Results

### CD103+CD8+ TRM has higher infiltration in CRC than in adjacent normal tissues

**Fig. 1:**
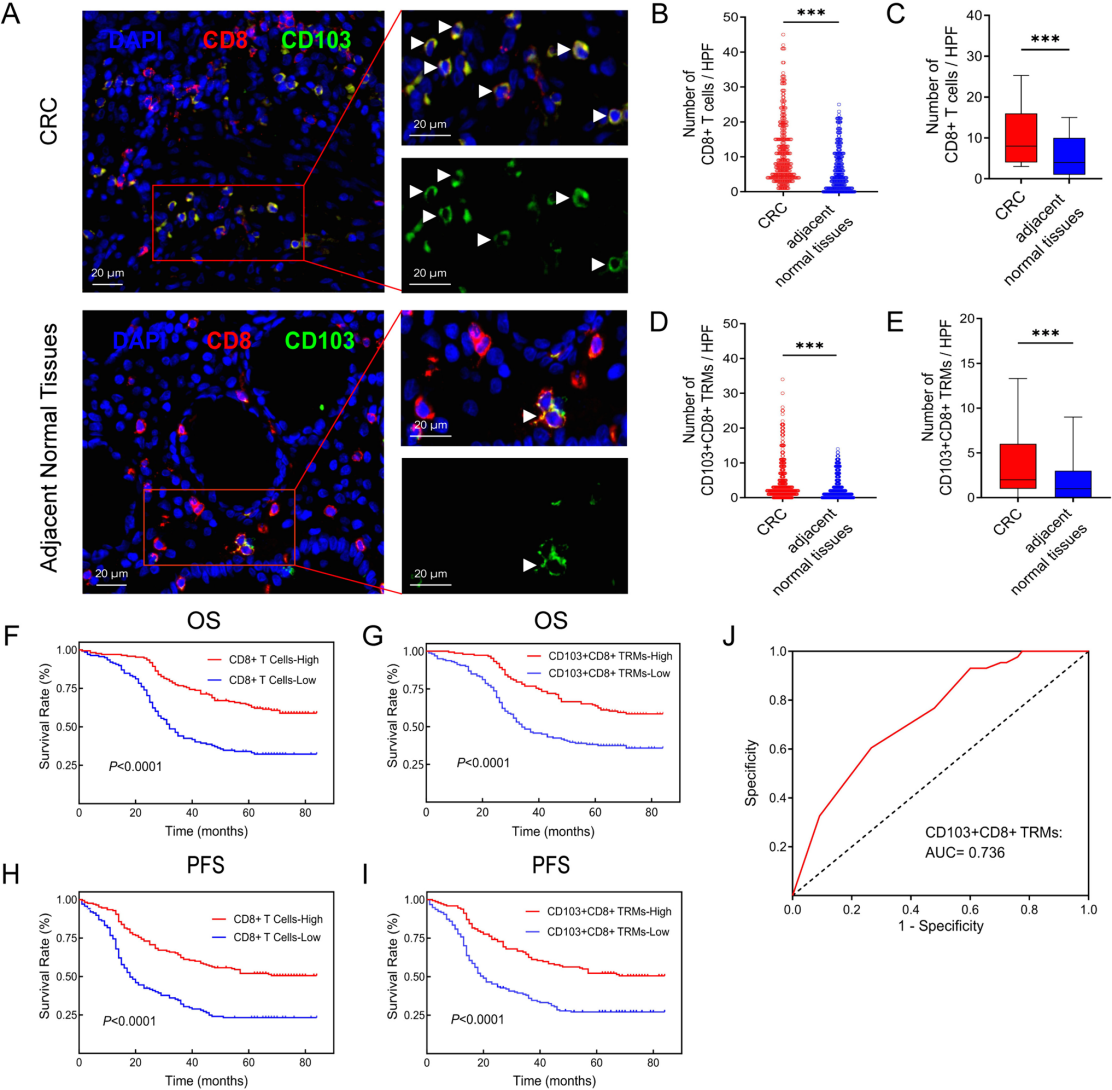
The number of CD8+ T cells and CD103+CD8+ TRMs in CRC and adjacent normal tissues and survival analysis according to their levels. **A** The CD103+CD8+ TRMs can be found in both CRC and adjacent normal tissues. **B**–**E** Comparison of the CD8+ T cells (**B** and **C**) and CD103+CD8+ TRMs (**D** and **E**) numbers in CRC and adjacent normal tissues. **F**–**I** The OS and PFS of CD8+ T cells (**F**, **H**) and the OS and PFS of CD103+CD8+ TRMs (**G**, **I**). **J** The ROC curve of CD103+CD8+ TRMs. (Immunofluorescent staining, ×400, the white triangle in the tissue is CD8+ TRM. HPF, high power field). CRC, colorectal cancer; OS, overall survival; PFS, progression-free survival; *TRM* Tissue-resident memory T cell. *p < 0.05; **p < 0.01; ***p < 0.001

First, we labeled T cells with CD8 and CD103 and observed the infiltration of CD8+ T cells and CD103+CD8+ TRMs in CRC and matched adjacent normal tissues using mIF. The results showed that the infiltration levels of CD8+ T cells and CD103+CD8+ TRMs were different between CRC and matched adjacent normal tissues (Fig. 1A). We further found that the number of CD8+ T cells and CD103+CD8+ TRMs in CRC tissues was higher than that in adjacent normal tissues (Fig. 1B–E). In summary, it was indicated that there were higher CD8+CD103+ TRMs infiltration levels in CRC tissues than in adjacent normal tissues.

### The correlation analysis between CD103+CD8+ TRMs infiltration level and clinicopathologic characteristics in CRC

Based on the above results, the cut-off value for the number of CD8+ T cells was 8/HPF and the number of CD103+CD8+ TRMs was 3/HPF calculated using X-tile software, and then the patients were divided into high-infiltration and low-infiltration groups. First, we found that highly infiltrated CD8+ T cells were correlated with a lower TNM stage and negative VEGF expression (*p* < 0.05; Table 1). Next, we explored the relationship between the infiltration level of CD103+CD8+ TRMs and the clinicopathological characteristics of patients with CRC. Similarly, the results showed that higher infiltration of CD103+CD8+ TRMs was associated with lower TNM stage and negative VEGF expression (*p* < 0.05; Table 1) but was not correlated with age, gender, tumor location, MMR status, and EGFR expression (*p* > 0.05; Table 1).

**Table 1:** Correlation between CD8+ T cells and CD103+CD8+ T cells and Clinicopathological Characteristics of CRC patients (*n* = 306)

Characteristics	Case (n)	Number of CD8+ T cell		χ^2^	*p-*value	Number of CD103+CD8+ TRM		χ^2^	*p-*value
		< 8	≥ 8			< 3	≥ 3		
*Age (y)*				0.747	0.387			2.891	0.089
< 60	104	43 (41.35)	61 (58.65)			47 (45.19)	57 (54.81)		
≥ 60	202	94 (46.53)	108 (53.47)			112 (55.45)	90 (44.55)		
*Gender*				0.002	0.968			3.394	0.065
Male	185	83 (44.86)	102 (55.14)			104 (56.22)	81 (43.78)		
Female	121	54 (44.63)	67 (55.37)			55 (45.45)	66 (54.55)		
*Location*				0.594	0.441			0.019	0.890
Left-side colon/Rectum	228	105 (46.05)	123 (53.95)			119 (52.19)	109 (47.81)		
Right-side colon	78	32 (41.03)	46 (58.97)			40 (51.28)	38 (48.72)		
*T-stage*				6.329	0.012			6.414	0.011
T_1_–T_2_	57	17 (29.82)	40 (70.18)			21 (36.84)	36 (63.16)		
T_3_–T_4_	249	120 (48.19)	129 (51.81)			138 (55.42)	111 (44.58)		
*N-stage*				19.710	< 0.001			12.368	< 0.001
N_0_	168	56 (33.33)	112 (66.67)			72 (42.86)	96 (57.14)		
N_1_–N_2_	138	81 (58.70)	57 (41.30)			87 (63.04)	51 (36.96)		
*M-stage*				20.684	< 0.001			12.311	< 0.001
M_0_	263	104 (39.54)	159 (60.46)			126 (47.91)	137 (52.09)		
M_1_	43	33 (76.74)	10 (23.26)			33 (76.74)	10 (23.26)		
*Clinical stage*				21.575	< 0.001			14.484	< 0.001
I–II	159	51 (32.08)	108 (67.92)			66 (41.51)	93 (58.49)		
III–IV	147	86 (58.50)	61 (41.50)			93 (63.27)	54 (36.73)		
*Neoadjuvant therapy*				3.616	0.057			2.351	0.125
Yes	43	25 (18.25)	18 (10.65)			27 (16.98)	16 (10.88)		
No	263	112 (81.75)	151 (89.35)			132 (83.02)	131 (89.12)		
*MMR status*				3.544	0.060			3.263	0.071
p-MMR	267	125 (46.82)	142 (53.18)			144 (53.93)	123 (46.07)		
d-MMR	39	12 (30.77)	27 (69.23)			15 (38.46)	24 (61.54)		
*EGFR expression*				0.064	0.801			0.047	0.828
Positive	210	93 (42.86)	117 (57.14)			110 (52.38)	100 (47.62)		
Negative	96	44 (48.96)	52 (51.04)			49 (51.04)	47 (48.96)		
*VEGF expression*				5.234	0.022			7.217	0.007
Positive	170	86 (50.59)	84 (49.41)			100 (58.82)	70 (41.18)		
Negative	136	51 (37.50)	85 (62.50)			59 (43.38)	77 (56.62)		
*KRAS mutant*				2.806	0.094			4.216	0.040
Positive	103	53 (38.69)	50 (48.54)			62 (38.99)	41 (27.89)		
Negative	203	84 (61.31)	119 (58.62)			97 (61.01)	106 (72.11)		
*NRAS mutant*				1.574	0.210			0.040	0.841
Positive	24	14 (9.93)	10 (6.06)			12 (7.55)	12 (8.16)		
Negative	282	127 (90.07)	155 (93.94)			147 (92.45)	135 (91.84)		
*BRAF mutant*				8.426	0.004			5.107	0.024
Positive	18	14 (10.22)	4 (2.37)			14 (8.81)	4 (2.72)		
Negative	288	123 (89.78)	165 (97.63)			145 (91.19)	143 (97.28)		

*MMR* mismatch repair, *d-MMR* different mismatch repair, *p-MMR* proficient mismatch repair, *EGFR* epidermal growth factor receptor, *VEGF* vascular endothelial growth factor, *TRM* tissue-resident memory T cell

### Highly infiltrated CD103+CD8+ TRMs predict a better prognosis in CRC

To investigate the correlation between CD8+ T cells and CD103+CD8+ TRMs and patient survival, Kaplan–Meier method and Cox proportional hazards analysis were performed. First, patients with highly infiltrated CD8+ T cells had longer survival times than those with low infiltration of CD8+ T cells (Fig. 1F, H). In addition, the death rate was lower in the CD103+CD8+ TRMs high-infiltration group than in the low-infiltration group (40.82% vs. 61.64%). We found that the OS and PFS in the CD103+CD8+ TRMs high-infiltration group were better than those in the low-infiltration group (Fig. 1G, I).

Next, the results of univariate analysis showed that the factors associated with OS in CRC patients were age, gender, clinical stage, EGFR expression, VEGF expression, and infiltration level of CD8+ T cells and CD103+CD8+ TRMs (*p* < 0.05), whereas tumor location and MMR status were not (*p* > 0.05). Furthermore, in the multifactorial analysis, the independent prognostic factors affecting OS in CRC patients were age, clinical stage, KRAS mutant, NRAS mutant, BRAF mutant, and infiltration level of CD103+CD8+ TRMs, while the remaining factors were not (Table 2). In summary, CRC patients with highly infiltrated CD103+CD8+ TRMs had a favorable prognosis, and the infiltration level of CD103+CD8+ TRMs was an independent prognostic factor.

**Table 2:** The univariate and multivariate Cox proportional hazards regression model in CRC patients (*n* = 306)

Characteristics	Univariate analysis		Multivariate analysis	
	HR (95%CI)	*p*-value	HR (95%CI)	*p*-value
Age (y) (≥ 60)	1.566 (1.105–2.218)	0.012	1.935 (1.350–2.773)	0.000
Gender (Male)	1.550 (1.112–2.162)	0.010	–	0.059
Location (Right-side colon)	1.104 (0.777–1.567)	0.582		
Clinical stage (III–IV)	4.843 (3.397–6.903)	0.000	4.461 (3.101–6.416)	0.000
Neoadjuvant therapy (Yes)	3.067 (2.106–4.467)	0.000	–	0.639
MMR status (d-MMR)	0.705 (0.492–1.009)	0.056	–	
EGFR expression (Positive)	1.496 (1.048–2.136)	0.027	–	0.231
VEGF expression (Positive)	1.505 (1.089–2.079)	0.013	–	0.061
KRAS mutant (Positive)	1.687 (1.227–2.319)	0.001	1.823(1.296–2.565)	0.001
NRAS mutant (Positive)	2.356 (1.471–3.775)	0.000	2.490(1.524–4.069)	0.000
BRAF mutant (Positive)	2.691 (1.551–4.668)	0.000	1.999(1.124–3.556)	0.018
CD8+ T cells infiltration (High)	0.412 (0.300–0.566)	0.000	–	0.138
CD103+CD8+ TRMs infiltration (High)	0.462 (0.334–0.638)	0.000	0.646 (0.464–0.900)	0.010

*CRC* colorectal cancer, *MMR* mismatch repair, *d-MMR* different mismatch repair, *p-MMR* proficient mismatch repair, *EGFR* epidermal growth factor receptor, *VEGF* vascular endothelial growth factor, *TRM* tissue-resident memory T cell

### The relationship between CD103+CD8+ TRMs and liver metastasis of CRC

In addition, when we divided the groups according to whether they had liver metastases, we surprisingly found that the infiltration level of CD103+CD8+ TRMs in CRC tissues appeared to be predictive of whether liver metastases occurred in CRC (when the cut-off value was 2/HPF, the sensitivity and specificity were 0.605 and 0.734, respectively) (Fig. 1J). In general, the results not only indicate that CD103+CD8+ TRMs have an independent influence on OS in CRC patients but also suggest that if we find CD8+ TRM < 2/HPF in CRC tissues in our clinical practice, it indicates that the patient may be more likely to develop liver metastasis. This may provide meaningful assistance in clinical diagnosis and treatment in the future. These results suggest that CD103+CD8+ TRMs could be used as new prognostic biomarkers.

### The infiltration level of CD103+CD8+ TRMs in CRC liver metastases is associated with EGFR and VEGF expression

**Fig. 2:**
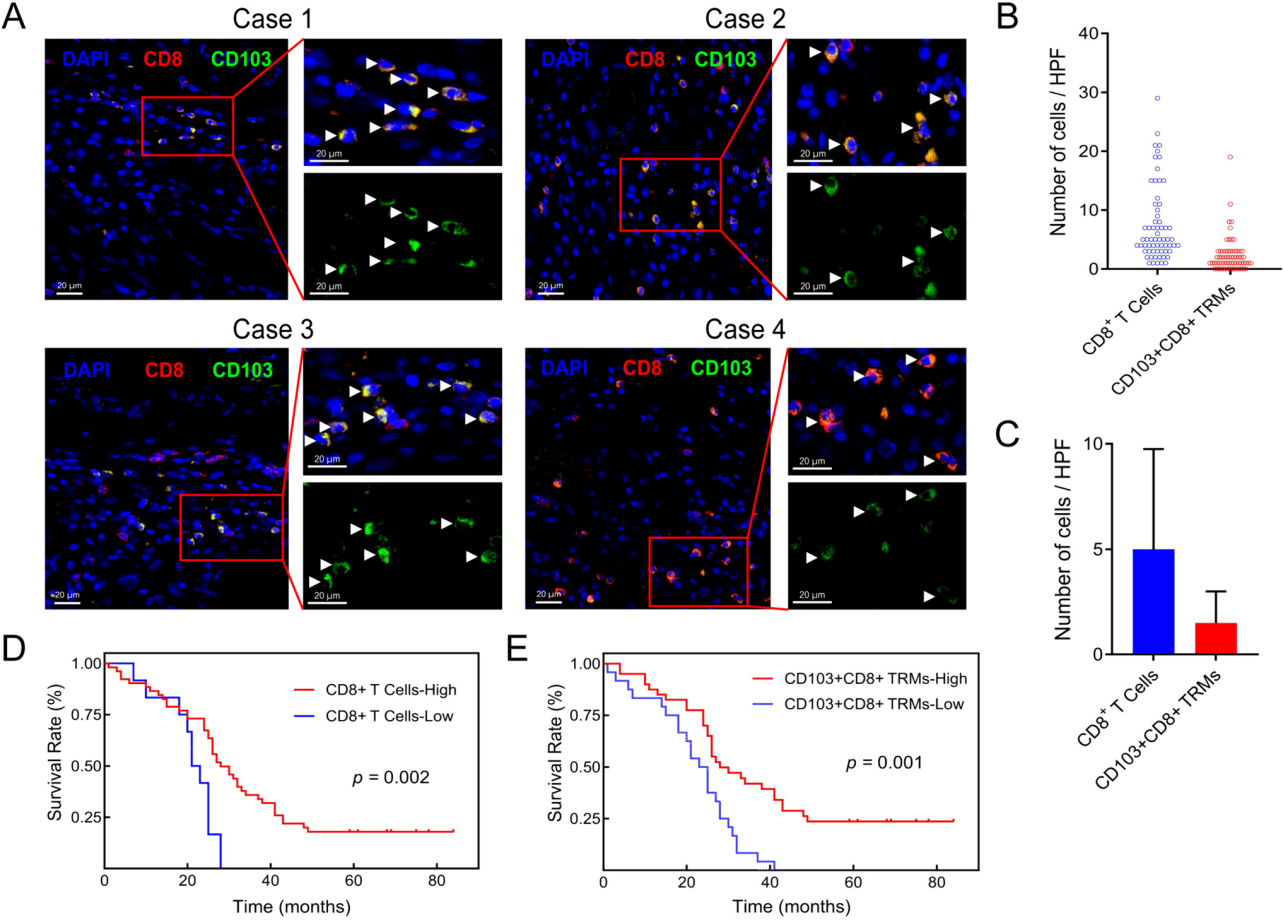
The infiltration level of CD8+ T cells and CD103+CD8+ TRMs in CRC liver metastasis and survival analysis according to their levels. **A** CD8+ T cells and CD103+CD8+ TRMs were shown in CRC liver metastasis by mIF. **B** and **C** The median and interquartile range of CD8+ T cells and CD103+CD8+ TRMs at HPF. (Immunofluorescent staining, × 400, The white triangle in the tissue is CD103+CD8+ TRMs). **D** and **E** The Kaplan–Meier analysis of CD8+ T cells (**D**) and CD103+CD8+ TRMs (**E**). OS, overall survival; *TRM* tissue-resident memory T cell

Based on these findings, we concluded that CD103+CD8+ TRMs in CRC tissues correlated with liver metastasis. Next, we investigated the clinical significance of CD103+CD8+ TRMs in liver metastasis of CRC. We obtained clinical data of patients with CRC liver metastasis (Supplementary Table 2) and evaluated the infiltration level of CD8+ T cells and CD103+CD8+ TRMs in CRC liver metastatic lesions (Fig. 2A–C). Interestingly, highly infiltrated CD8+ T cells and CD103+CD8+ TRMs were both significantly associated with negative EGFR and VEGF expression, and CD103+CD8+ TRMs were also associated with MMR status in patients with CRC liver metastases (Table 3). So, combined with previous results, the infiltration level of CD103+CD8+ TRMs might be connected to tumor vascular factors (e.g., VEGF) and tumor immunogenicity.

**Table 3:** Correlation between CD8+ T cells and CD103+CD8+ T cells and clinicopathological characteristics of CRC patients with liver metastases (*n* = 64)

Characteristics	Case (n)	Number of CD8+ T cell		χ^2^	*p* value	Number ofCD103+CD8+ TRM		χ^2^	*p* value
		< 4	≥ 4			< 3	≥ 3		
*Age* (*y*)				2.053	0.152			3.122	0.077
< 60	22	2 (9.09)	20 (90.91)			5 (22.73)	17 (77.27)		
≥ 60	42	10 (23.81)	32 (76.19)			19 (45.24)	23 (54.76)		
*Gender*				0.020	0.887			0.145	0.703
Male	49	9 (18.37)	40 (81.63)			19 (38.78)	30 (61.22)		
Female	15	3 (20.00)	12 (80.00)			5 (33.33)	10 (66.67)		
*Location*				0.379	0.538			0.109	0.741
Left-side colon/Rectum	52	9 (17.31)	43 (82.69)			19 (36.54)	33 (63.46)		
Right-side colon	12	3 (25.00)	9 (75.00)			5 (41.67)	7 (58.33)		
*Neoadjuvant therapy*				0.234	0.628			0.068	0.795
Yes	28	6 (21.43)	22 (78.57)			11 (39.29)	17 (60.71)		
No	36	6 (16.67)	30 (83.33)			13 (36.11)	23 (63.89)		
*MMR status*				1.528	0.216			3.972	0.046
p-MMR	58	12 (20.69)	46 (79.31)			24 (41.38)	34 (58.62)		
d-MMR	6	0 (0.00)	6 (100.00)			0 (0.00)	6 (100.00)		
*EGFR expression*				4.135	0.042			4.120	0.042
Positive	50	12 (24.00)	38 (76.00)			22 (44.00)	28 (56.00)		
Negative	14	0 (0.00)	14 (100.00)			2 (14.29)	12 (85.71)		
*VEGF expression*				6.940	0.008			4.651	0.031
Positive	37	11 (29.73)	26 (70.27)			18 (48.65)	19 (51.35)		
Negative	27	1 (3.70)	26 (96.30)			6 (22.22)	21 (77.78)		
*KRAS mutant*				0.026	0.872			0.068	0.795
Positive	28	5 (17.86)	23 (82.14)			11 (39.29)	17 (60.71)		
Negative	36	7 (19.44)	29 (80.56)			13 (36.11)	23 (63.89)		
*NRAS mutant*				0.006	0.941			0.145	0.904
Positive	5	1 (20.00)	4 (80.00)			2 (40.00)	3 (60.00)		
Negative	59	11 (18.64)	48 (81.36)			22 (37.29)	37 (62.71)		
*BRAF mutant*				2.110	0.146			0.610	0.435
Positive	8	3 (37.50)	5 (62.50)			4 (50.00)	4 (50.00)		
Negative	56	9 (16.07)	47 (83.93)			20 (35.71)	36 (64.29)		

*MMR* mismatch repair, *d-MMR* different mismatch repair, *p-MMR* proficient mismatch repair, *EGFR* epidermal growth factor receptor, *VEGF* vascular endothelial growth factor, *TRM* tissue-resident memory T cell

### CD103+CD8+ TRMs in CRC liver metastasis were associated with patients' prognosis

Based on the infiltration level of CD103+CD8+ TRMs in CRC liver metastasis, Kaplan–Meier analysis revealed a significant prolongation of OS in patients in the CD8+ TRM high-infiltration group (Fig. 2D, E). Furthermore, using the Cox risk regression model, we found that the independent prognostic factors affecting OS in patients with CRC liver metastases were EGFR expression and infiltration level of CD103+CD8+ TRMs (Supplementary Table 3). In summary, highly infiltrated CD103+CD8+ TRMs predicted better prognosis in CRC and CRC liver metastasis. More importantly, CRC patients with a high-infiltration level of CD103+CD8+ TRMs were less likely to develop liver metastasis than those with low infiltration.

### Anti-angiogenic therapy promotes infiltration of CD103+CD8+ TRMs in tumor

**Fig. 3:**
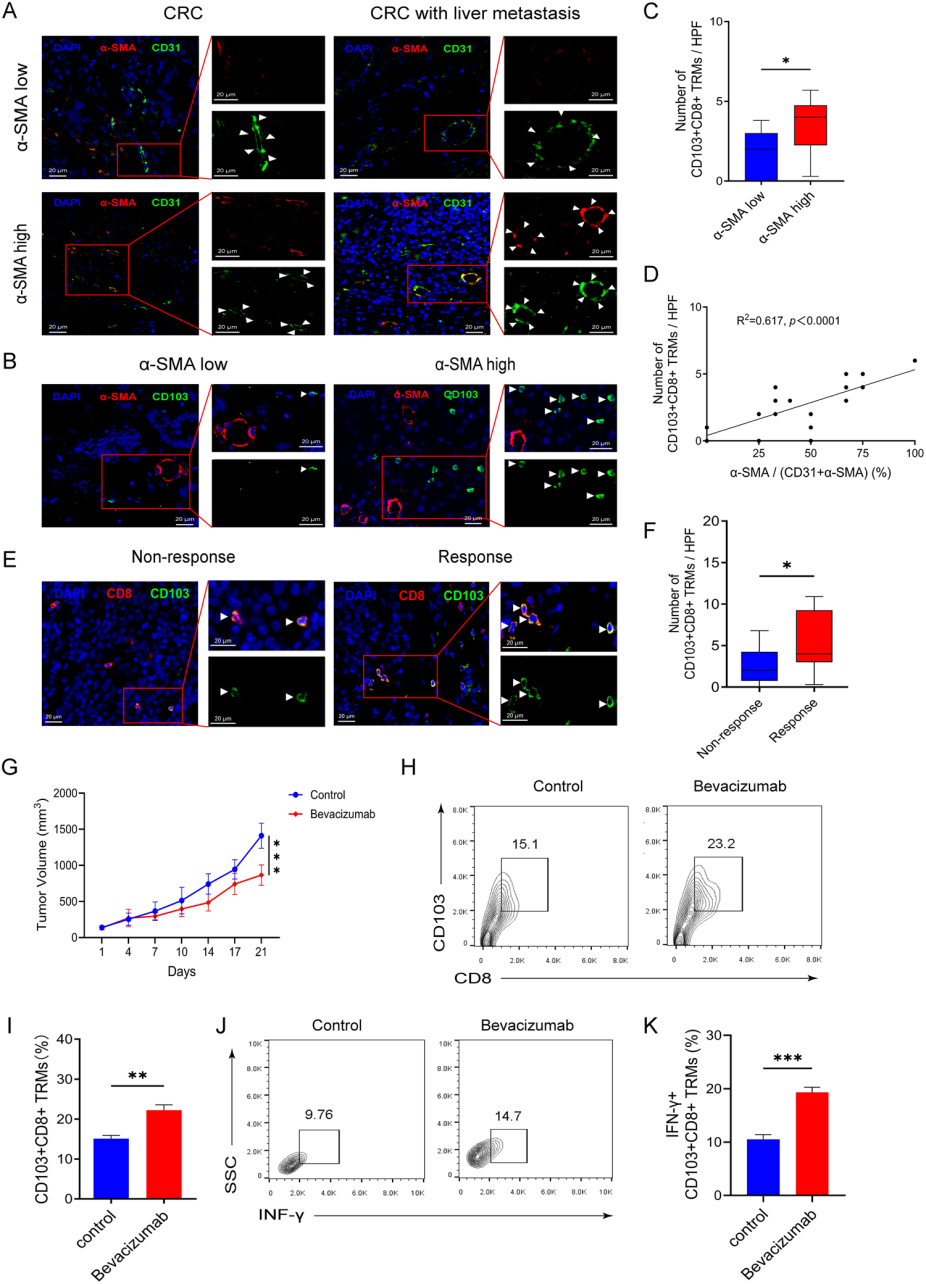
The expression of CD31 and α-SMA in clinical samples and the anti-angiogenic therapy promotes infiltration of CD103+CD8+ TRMs in tumor. **A** The expression of CD31 and α-SMA in CRC and CRC liver metastasis tissue. **B** The infiltration level of CD103+CD8+ TRMs in the α-SMA high and low groups. **C** Comparison of median number and interquartile range of CD103+CD8+ TRMs. **D** The relationship of CD103+CD8+ TRMs and α-SMA+ vessel. **E**–**F** The infiltration level of CD103+CD8+ TRMs in CRC patients who responded and non-respond to bevacizumab treatment. **G** Average tumor growth curves showing tumor volume in mice treated with either control or the Bevacizumab (*n* = 5 mice per group). **H**–**K** Representative flow cytometry plots (**H**) and the percentage of CD103+CD8+ TRMs (**I**) and representative flow cytometry plots (J) and the percentage of IFN-γ+ CD103+CD8+ TRMs (K) isolated from mice on day 21 (*n *= 5 mice per group). (Immunofluorescent staining, × 400, The white triangle in the tissue is CD103+CD8+ TRMs). CRC, colorectal cancer; TRM, tissue-resident memory T cell. α-SMA, alpha-smooth muscle actin. *p < 0.05; **p < 0.01; ***p < 0.001

In this study, we found that the infiltration level of CD103+CD8+ TRMs is related to VEGF expression (Tables 1 and 3). Therefore, we labeled and counted α-SMA and CD31 in clinical samples, and the results showed that different proportions of α-SMA+ vessels and/or CD31+ vessels were expressed in the tissues (Fig. 3A). We categorized patients into high and low α-SMA expression groups based on the median ratio (50%) of α-SMA+ vessels to the sum of CD31+ and/or α-SMA+ vessels (α-SMA/[CD31+α-SMA]) as the cut-off value. We found a higher infiltration level of CD103+CD8+ TRMs in the α-SMA high expression group (Fig. 3B, C). Correlation analysis was also performed. The results indicated a significant positive correlation between the infiltration level of CD103+CD8+ TRMs and the ratio of [α-SMA/(CD31+α-SMA)] (Fig. 3D). Meanwhile, validated by clinical tissue samples, we also found that the infiltration level of CD103+CD8+ TRMs was higher in patients who responded to bevacizumab treatment (Fig. 3E, F). Therefore, we further established a C57BL/6 mouse tumor model, and the results showed that, compared with the control group, the bevacizumab group had a smaller tumor volume, a higher level of infiltration of CD103+CD8+ TRMs in the tumor tissues, and a higher tumor-killing ability (Fig. 3G–K). This implies that CD103+CD8+ TRMs are associated with the density of normal blood vessels in tumor and with the efficacy of anti-tumor angiogenesis therapy in clinical practice.

### CD103+CD8+ TRMs have significant anti-tumor ability

**Fig. 4:**
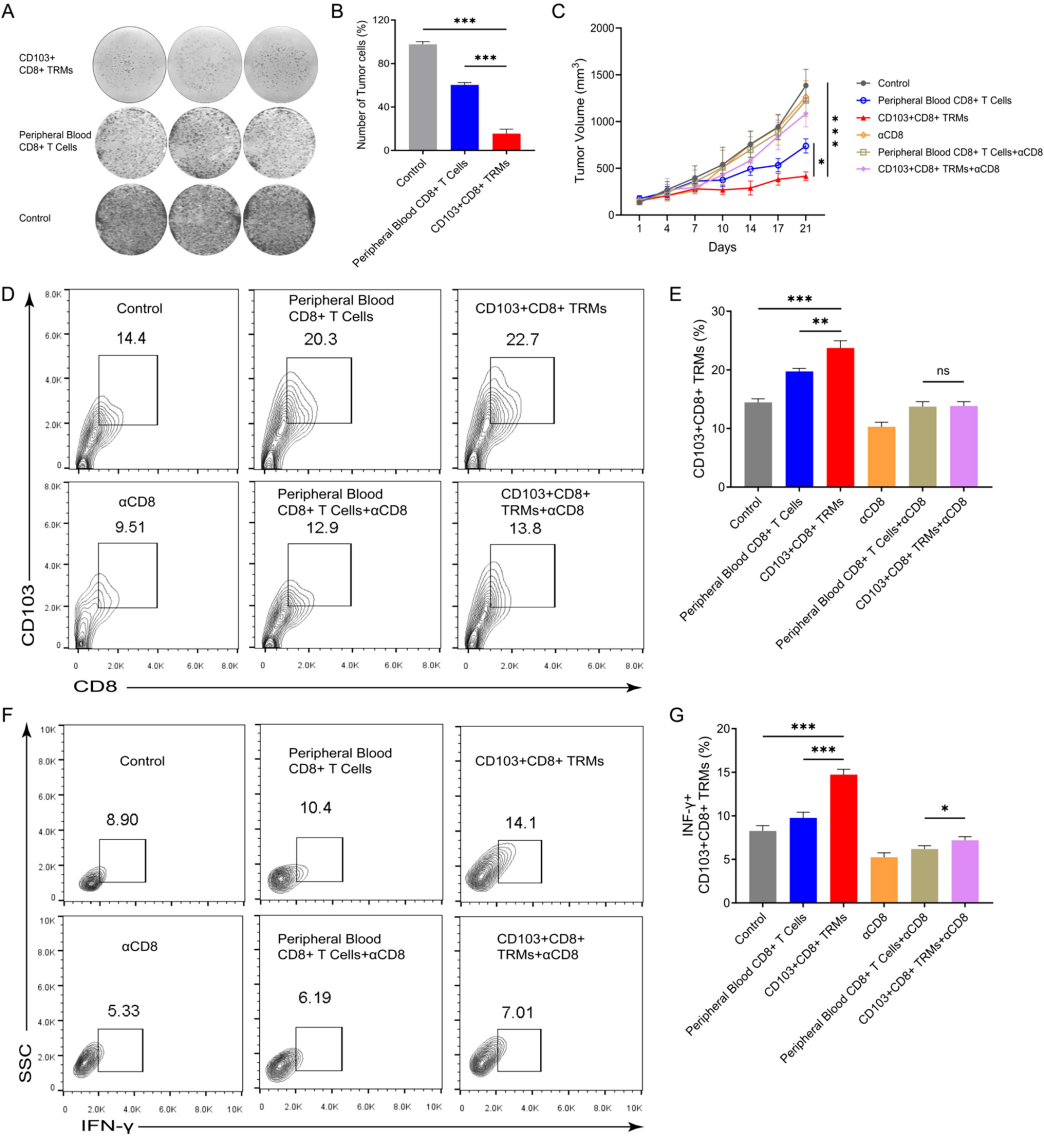
The CD103+CD8+ TRMs have significant anti-tumor ability. **A** and **B** The colony-formation assays (**A**) and quantifiable results (**B**) of different interventions on tumor cells were analyzed. **C** Average tumor growth curves showing tumor volume in mice treated with different interventions (*n* = 5 mice per group). A two-way ANOVA with Sidak’s multiple comparison test was performed. **D** and **E.** Representative flow cytometry plots (**D**) and the percentage of CD103+CD8+ TRMs (**E**) isolated from mice on day 21 (*n* = 5 mice per group). **F** and **G.** Representative flow cytometry plots (**F**) and the percentage of IFN-γ+ CD103+CD8+ TRMs (**G**) isolated from mice on day 14 (*n* = 5 mice per group). Two-tailed unpaired Student’s t test was performed. *p < 0.05; **p < 0.01; ***p < 0.001

Next, we investigated the anti-tumor ability of CD103+CD8+ TRMs. We found that CD103+CD8+ TRMs were more effective than CD8+ T cells from peripheral blood in killing tumors when co-cultured with tumor cells in vitro (Fig. 4A, B). Meanwhile, we cultured the extracted CD103+CD8+ TRMs and then passed them through adoptive therapy and found that the tumor volume was smaller in the CD103+CD8+ TRMs treatment group (Fig. 4C). A significant difference was observed in CD103+CD8+ TRMs infiltrating the tumor compared to the peripheral blood CD8+ T cell group, and the killing ability of the CD103+CD8+ TRMs treatment group was greater (Fig. 4D–F). This also suggests that CD103+CD8+ TRMs have good prospects for clinical applications.

## Discussion

Worldwide, the morbidity and mortality rates of CRC remain high, and its prognosis remains unsatisfactory [2, 3]. Immune checkpoint inhibitors (ICIs) have achieved remarkable efficacy in clinical practice and are widely used to treat a variety of tumors, including melanoma, lung cancer, and CRC. Regrettably, only a small percentage of patients with CRC benefit from immunotherapy [18]. High heterogeneity within the TME may be one of the most important factors. In general, we consider that CD8+ T cells in the TME can regain their anti-tumor effects because they can respond to ICIs targeting pathways such as PD-1/PD-L1. However, not all CD8+ T cells respond to ICIs because they are highly heterogeneous in the TME [19]. Therefore, it is important to elucidate the subpopulation of CD8+ T cells and their role in the TME. As a specific subpopulation of memory T cells, CD103+CD8+ TRMs play an essential role in immune monitoring and responses of the body. However, the relationship between the infiltration level of CD103+CD8+ TRMs and clinicopathological characteristics and prognosis of CRC, and the potential for clinical applications of CD103+CD8+ TRMs remains unclear. In this study, we retrospectively analyzed the clinical data of 306 CRC patients who attended the First Affiliated Hospital of Jinan University and assessed the relationship between the infiltration level of CD103+CD8+ TRMs and clinicopathological characteristics and prognosis of CRC patients. The anti-tumor ability and clinical application potential of CD103+CD8+ TRMs were elucidated by in vitro and in vivo experiments.

In this study, we demonstrated the relationship between the infiltration level of CD103+CD8+ TRMs and clinicopathological characteristics, such as TNM stage, VEGF expression, KRAS mutant, and BRAF mutant in CRC. Our results found that the infiltration level of CD103+CD8+ TRMs is lower in patients with KRAS and BRAF mutations, which may also contribute to the poor prognosis of such patients. Similarly, Toh et al. [20] showed that MSI-H patients had higher of infiltration level of CD103+CD8+ TRMs than MSS patients, but there was no significant difference between the groups of patients with BRAF mutant and the BRAF wild-type. The reasons for this may be the small sample size included in the study and the fact that the immune background of these populations was all patients with MSI-H. In another study, a significant increase in the frequency of CD103+CD8+ TRMs was found after trametinib treatment in a BRAF-mutated PDX mouse model [21]. In addition, KRAS mutant promotes metabolic reprogramming, which generates nutrients necessary to maintain unrestricted proliferation of tumor cells [22]. Thus, in KRAS-mutant tumors, there may be a more pronounced immunosuppressive microenvironment that affects the residency and proliferation of CD103+CD8+ TRMs. Overall, this also implies that CD103+CD8+ TRMs may be associated with the status of mutant genes such as KRAS and BRAF. However, it is still necessary for further investigation to elucidate how mutant genes such as KRAS and BRAF affect the infiltration of CD103+CD8+ TRMs.

In addition, the highly infiltrated CD103+CD8+ TRMs predicted a longer OS in CRC patients. Interestingly, in demonstrating that CD103+CD8+ TRMs are an independent prognostic factor affecting OS in CRC, we also found that the infiltration level of CD103+CD8+ TRMs in CRC tissues was predictive of liver metastases in CRC. This result implies a higher infiltration level of CD103+CD8+ TRMs in CRC tissues, which are the least likely to develop liver metastasis. In general, our study showed that CD103+CD8+ TRMs are valid prognostic biomarkers and that high infiltration is an independent factor influencing the favorable prognosis of CRC patients. Meanwhile, some studies have found that CD103+CD8+ TRMs are positively correlated with OS in patients with esophageal squamous carcinoma, which has an effective anti-tumor effect after immune checkpoint blockers (ICBs) and is not affected by chemotherapy [23]. In addition, CD103+CD8+ TRMs can highly express immune checkpoint molecules and effector proteins and positively correlate with patient OS and DFS in breast cancer [16]. Moreover, some studies have found that highly infiltrated CD8+ TRM improves the prognosis of patients with cervical cancer, and that HPV E6/E7 vaccine therapy combined with radiotherapy significantly increases the number of intratumoral CD103+CD8+ TRMs [24].These findings imply a close relationship between TRM and immunotherapy and may explain part of the mechanism of radiotherapy (stereotactic radiotherapy) sensitizing immunotherapy. Thus, CD103+CD8+ TRMs may have great potential for clinical applications in the future.

In recent years, with the in-depth study of the TME, it has been found that some inflammatory mediators or cytokines in the TME play an important role in the development and infiltration of CD103+CD8+ TRMs. In this process, the cytokines such as TGF-β, IL-15 and IL-33 play an important role, for example, TGF-β promotes TRM formation by down-regulating Eomes and T-bet [25]. And IL-33 promotes TRM formation by inhibiting KLF2 and down-regulating S1PR1 via PI3K/AKT pathway [26]. In addition, Bhlhe40 has been shown to be a key regulator of CD8+ TRM formation and function, as deletion of Bhlhe40 significantly reduces CD8+ TRM formation and attenuates the anti-tumor activity of CD8+ T cells [27]. Researchers have also shown that the lifespan of CD8+ T cells is closely linked to fatty acid-binding protein 4/5 (Fabp4/Fabp5) [28, 29].

VEGF is one of the strongest angiogenesis-stimulating factors and closely associated with tumor angiogenesis [30]. However, the relationship between anti-angiogenic therapy or VEGF expression and TRM has not been reported. In this study, we found that the infiltration level of CD103+CD8+ TRMs correlated with VEGF expression in both CRC and CRC patients with liver metastasis. Therefore, when we further investigated the relationship between CD8+ TRM and tumor vasculature, the results showed that the infiltration level of CD103+CD8+ TRMs was correlated with the expression of α-SMA, and its infiltration was positively correlated with the density of α-SMA + vessels. More importantly, we demonstrated that anti-angiogenic therapy could increase the infiltration level of CD103+CD8+ TRMs in tumor. Meanwhile, the expression of a variety of cytokines such as CXCL10 can be inhibited by pro-angiogenic factors [31]. Thus, once the tumor vasculature is normalized, this inhibitory effect may be eliminated, allowing more cytotoxic T and NK cells to infiltrate the tumor tissue, which may include TRM. Furthermore, Kim et al. [32] showed that the degree of CD69+CD103+CD8+ TRM infiltration was positively correlated with the expression of inflammatory T cell markers (e.g., E-selectin, CXCL11 and CXCL13) and that anti-angiogenic treatment could modulate the levels of the above cytokines. This also suggests that anti-angiogenic treatment may be able to promote the infiltration of CD8+ TRM. Unterleuthner et al. [33] showed that WNT2 activates the typical Wnt/β-catenin signaling pathway and upregulates proteins associated with pro-angiogenic function, so promoting the development of colorectal cancer and angiogenesis. Meanwhile, some signaling pathways such as Wnt/β-catenin and TGF-β were found to be enriched in tumors with low TRM infiltration [32]. Thus, the low level of infiltration of the TRM may be closely related to the aberrant angiogenesis of the tumor. And anti-angiogenic therapy improves TME and increases the infiltration level of CD103+CD8+ TRMs. More importantly, we also discovered that T cells of the CD103+CD8+ TRMs subpopulation were more advantageous in tumor killing compared to regular peripheral blood CD8+ T cells. This may be related to its resident properties or organ specificity, making it more adaptable to the TME and the recognition of tumor antigens [34]. This also suggests that it is possible to achieve the effect of “1 + 1 > 2” when combined with immunotherapy. In addition, CD103+CD8+ TRMs can be used to benefit more patients through adoptive cell transfer therapy (ACT).

However, our study also has some limitations, such as the specific mechanism by which anti-tumor angiogenesis therapy promotes infiltration of CD103+CD8+ TRMs still needs to be further explored. And many studies have shown that CD103 is a characteristic marker for CD8+ TRMs, but there may still be some bias. Nonetheless, we show that CD103+CD8+ TRMs are inextricably linked to the normalization of tumor vasculature. This also provides some evidence for follow-up research and the combined application strategy of anti-tumor angiogenesis therapy (especially combined immunotherapy strategy).

In conclusion, we discovered the role of CD103+CD8+ TRMs in CRC and their association with liver metastasis and tumor vessels. And we identified a novel biomarker for assessing the risk and prognosis of liver metastasis in CRC patients, which provides a reference for more accurate and timely individualized treatment plans for CRC liver metastatic patients.

### Supplementary Information

Below is the link to the electronic supplementary material.

Supplementary file1 (DOC 95 KB)

Supplementary file2 (DOC 88 KB)

## Data Availability

The original contributions presented in the study are included in the article/supplementary material. Further inquiries can be directed to the corresponding authors.
