# Supplementary material for: Tissue-resident memory CD103+CD8+ T cells in colorectal cancer: its implication as a prognostic and predictive liver metastasis biomarker
Source: Cancer Immunol Immunother. 2024 Jul 2;73(9):176. doi: 10.1007/s00262-024-03709-2 (PMC11219596; doi:10.1007/s00262-024-03709-2)
Supplement: Supplementary file 1 — Supplementary file1 (DOC 95 KB) [file 262_2024_3709_MOESM1_ESM.doc]

**Tissue-resident memory CD103+CD8+ T cells in colorectal cancer: Its implication as a prognostic and predictive liver metastasis biomarker**

Shijin Liu^1†^, Penglin Wang^2†^, Peize Wang^1†^, Zhan Zhao^1^, Xiaolin Zhang^3*^, Yunlong Pan^1,4*^, Jinghua Pan^1*^

**Supplementary Material**

**Supplementary Table 1. The Clinicopathological Characteristics of CRC Patients (n=306)**

| **Characteristic** | **Number of cases [n (%)]** |
| --- | --- |
| **Age（y）** |  |
| < 60 | 104 (33.99) |
| ≥ 60 | 202 (66.01) |
| **Gender** |  |
| Male | 185 (60.46) |
| Female | 121 (39.54) |
| **Location** |  |
| Left-side colon/ rectum | 228 (74.51) |
| Right-side colon | 78 (25.49) |
| **T stage** |  |
| T_1_-T_2_ | 57 (18.63) |
| T_3_-T_4_ | 249 (81.37) |
| **N stage** |  |
| N_0_ | 168 (54.90) |
| N_1_-N_2_ | 138 (45.10) |
| **M stage** |  |
| M_0_ | 263 (85.95) |
| M_1_ | 43 (14.05) |
| **Clinical stage** |  |
| I-II | 159 (51.96) |
| III-IV | 147 (48.04) |
| **Neoadjuvant therapy** |  |
| Yes | 43(14.05) |
| No | 263 (85.95) |
| **MMR status** |  |
| p-MMR | 267 (87.25) |
| d-MMR | 39 (12.75) |
| **EGFR expression** |  |
| Positive | 210 (68.63) |
| Negative | 96 (31.37) |
| **VEGF expression** |  |
| Positive | 170 (55.56) |
| Negative | 136 (44.44) |
| **KRAS-mutant** |  |
| Positive | 103 (33.66) |
| Negative | 203 (66.34) |
| **NRAS-mutant** |  |
| Positive | 24 (7.84) |
| Negative | 282 (92.16) |
| **BRAF-mutant** |  |
| Positive | 18 (5.88) |
| Negative | 288 (94.12) |
| **CD8+T cells infiltration** |  |
| High | 169 (55.23) |
| Low | 137 (44.77) |
| **CD103+CD8+ TRMs infiltration** |  |
| High | 147 (48.04) |
| Low | 159 (51.96) |

CRC, colorectal cancer; MMR, mismatch repair; d-MMR, different Mismatch Repair; p-MMR, proficient mismatch repair; EGFR, epidermal growth factor receptor; VEGF, vascular endothelial growth factor; TRM, Tissue-resident memory T cell.

# Supplementary Table 2. The Clinicopathological Characteristics of CRC Patient with liver metastases (n=64).

| **Characteristic** | **Number of cases [n (%)]** |
| --- | --- |
| **Age（y）** |  |
| < 60 | 22(34.38) |
| ≥ 60 | 42(65.62) |
| **Gender** |  |
| Male | 49(76.56) |
| Female | 15(23.44) |
| **Location** |  |
| Left-side colon/ Rectum | 52(81.25) |
| Right-side colon | 12(18.75) |
| **MMR status** |  |
| p-MMR | 58(90.63) |
| d-MMR | 6(9.37) |
| **EGFR expression** |  |
| Positive | 50(78.13) |
| Negative | 14(21.87) |
| **VEGF expression** |  |
| Positive | 37(57.81) |
| Negative | 27(42.19) |
| **CD8+ T cell infiltration** |  |
| High | 52(81.25) |
| Low | 12(18.75) |
| **CD103+CD8+ TRMs infiltration** |  |
| High | 40(62.50) |
| Low | 24(37.50) |

CRC, colorectal cancer; MMR, mismatch repair; d-MMR, different Mismatch Repair; p-MMR, proficient mismatch repair; EGFR, epidermal growth factor receptor; VEGF, vascular endothelial growth factor; TRM, Tissue-resident memory T cell.
