# Supplementary material for: Tissue-resident memory CD103+CD8+ T cells in colorectal cancer: its implication as a prognostic and predictive liver metastasis biomarker
Source: Cancer Immunol Immunother. 2024 Jul 2;73(9):176. doi: 10.1007/s00262-024-03709-2 (PMC11219596; doi:10.1007/s00262-024-03709-2)
Supplement: Supplementary file 2 — Supplementary file2 (DOC 88 KB) [file 262_2024_3709_MOESM2_ESM.doc]

**Tissue-resident memory CD103+CD8+ T cells in colorectal cancer: Its implication as a prognostic and predictive liver metastasis biomarker**

Shijin Liu^1†^, Penglin Wang^2†^, Peize Wang^1†^, Zhan Zhao^1^, Xiaolin Zhang^3*^, Yunlong Pan^1,4*^, Jinghua Pan^1*^

**Table 4** The univariate and multivariate Cox proportional hazards regression model in the CRC patients with liver metastases (n=64)

| **Characteristics** | **Univariate analysis** | | |  | | **Multivariate analysis** | | | |  |
| --- | --- | --- | --- | --- | --- | --- | --- | --- | --- | --- |
|  | **HR（95%CI）** | ***p*-value** | |  | | **HR（95%CI）** | | ***p*-value** | |  |
| Age (y) (≥60) | 1.196  （0.684-2.092） | | 0.531 | |  | |  | |  | |
| Gender (Male) | 1.290  （0.663-2.508） | | 0.453 | |  | |  | |  | |
| Location  (Right-side colon) | 1.522  （0.796-2.911） | | 0.204 | |  | |  | |  | |
| Neoadjuvant therapy  (Yes) | 1.422  （0.831-2.434） | | 0.199 | |  | |  | |  | |
| MMR status  (d-MMR) | 0.651  （0.234-1.805） | | 0.409 | |  | |  | |  | |
| EGFR expression  (Positive) | 4.086  （1.830-9.124） | | 0.001 | |  | | 3.655  （1.620-8.243） | | 0.000 | |
| VEGF expression  (Positive) | 1.626  （0.924-2.859） | | 0.092 | |  | |  | |  | |
| KRAS-mutant  (Positive) | 1.442  （0.843-2.466） | | 0.181 | |  | |  | |  | |
| NRAS-mutant  (Positive) | 1.31  （0.520-3.300） | | 0.566 | |  | |  | |  | |
| BRAF-mutant  (Positive) | 2.189  （0.969-4.944） | | 0.059 | |  | |  | |  | |
| CD8+ T cells infiltration (High) | 0.351  （0.174-0.706） | | 0.003 | |  | | - | | 0.476 | |
| CD103+CD8+ TRMs infiltration (High) | 0.390  （0.219-0.693） | | 0.001 | |  | | 0.458  （0.254-0.825） | | 0.010 | |

MMR, mismatch repair; d-MMR, different mismatch repair; p-MMR, proficient mismatch repair; EGFR, epidermal growth factor receptor; VEGF, vascular endothelial growth factor; TRM, Tissue-resident memory T cell.
